# Serum adropin levels and their association with sarcopenia in patients with diabetic nephropathy: a cross-sectional study

**DOI:** 10.3389/fendo.2026.1884793

**Published:** 2026-06-24

**Authors:** Emel Tatlı, Tuğba Kip

**Affiliations:** 1 Department of Nephrology, University of Health Sciences, Gaziosmanpaşa Training and Research Hospital, Istanbul, Türkiye; 2 Independent Researcher, Istanbul, Türkiye; 3 Medical Department, Abdi İbrahim Pharmaceuticals, Istanbul, Türkiye

**Keywords:** adropin, diabetic nephropathy, EWGSOP2, hand-grip strength, sarcopenia, total antioxidant status, type 2 diabetes mellitus

## Abstract

**Background:**

Adropin, an endogenous peptide hormone with established roles in energy homeostasis and endothelial function, has been implicated in metabolic disease and chronic kidney disease. However, its relationship with sarcopenia in diabetic nephropathy (DN), an increasingly recognised complication of type 2 diabetes mellitus (T2DM), remains poorly characterised. We evaluated serum adropin in T2DM patients with and without DN compared with a younger healthy control group (with the age difference acknowledged as a major confounder) and assessed whether adropin levels are associated with sarcopenia indices and total antioxidant status (TAS).

**Methods:**

This cross-sectional study included 83 participants: 59 patients with T2DM (37 with DN, 21 without DN (1 with missing UACR)) and 24 healthy controls. Sarcopenia evaluation, including body composition by bioelectrical impedance analysis and hand-grip strength using a hand dynamometer, was completed in 31 diabetic patients. Sarcopenia was operationally defined by reduced hand-grip strength according to EWGSOP2 thresholds (<27 kg for men, <16 kg for women). Serum adropin was measured by ELISA; TAS and TOS were measured by the Erel colorimetric method.

**Results:**

Serum adropin was significantly lower in T2DM patients than in controls (197.4 ± 129.1 vs 352.3 ± 240.7 pg/mL, p = 0.011). Within the diabetic cohort, adropin did not differ between DN and non-DN patients (201.4 ± 119.7 vs 193.8 ± 139.1 pg/mL, p = 0.370; ROC AUC 0.574). In the sarcopenia subgroup (n = 31), hand-grip strength was significantly lower in DN than non-DN patients (1st measurement: 18.7 ± 4.7 vs 26.5 ± 9.5 kg, p = 0.012; 3rd measurement: 17.8 ± 4.8 vs 25.1 ± 9.1 kg, p = 0.035), while muscle mass and walking speed did not differ. Patients meeting EWGSOP2 grip criteria for low strength showed numerically lower adropin than non-sarcopenic patients (169.5 ± 73.6 vs 238.9 ± 181.7 pg/mL, p = 0.358). Adropin correlated strongly with TAS (ρ = 0.82, p < 0.001).

**Conclusion:**

Serum adropin is markedly reduced in T2DM but does not discriminate DN from non-DN patients in this cohort. Reduced hand-grip strength is the most consistent sarcopenia-related abnormality in DN patients, supporting a functional rather than purely anthropometric muscle deficit. The strong positive correlation between adropin and TAS suggests that adropin tracks the broader oxidative-metabolic milieu of T2DM rather than functioning as an independent clinical biomarker for DN or sarcopenia; longitudinal validation is required before any biomarker role can be considered.

## 1 Introduction

Diabetic nephropathy (DN) remains the leading cause of chronic kidney disease (CKD) progression and end-stage renal disease worldwide, affecting approximately one-third of patients with type 2 diabetes mellitus (T2DM) over their lifetime (1). Beyond its established cardiovascular and renal complications, DN is increasingly recognised as a driver of secondary sarcopenia—the progressive loss of skeletal muscle mass and function—through chronic inflammation, insulin resistance, uraemic toxin accumulation, and reduced physical activity (2, 3). Sarcopenia in CKD is independently associated with frailty, hospitalisation, cardiovascular events, and mortality, making its early identification and biomarker-based stratification a priority for nephrology research (4).

Adropin (Energy Homeostasis Associated, ENHO gene) is a 76-amino-acid peptide hormone secreted predominantly by the liver and brain, with downstream actions on energy expenditure, glucose homeostasis, endothelial nitric-oxide synthesis, and insulin sensitivity (5, 6). Lower circulating adropin has been described in obesity, metabolic syndrome, T2DM, and atherosclerotic cardiovascular disease (7, 8), and recent reports describe associations with CKD progression and dialysis outcomes (9, 10). Mechanistically, adropin facilitates VEGFR-2 signalling, supports endothelial barrier function, and modulates oxidative-stress responses, all of which are relevant to both microvascular renal injury and skeletal-muscle anabolic resistance (11, 12).

The intersection between adropin biology and sarcopenia has emerged more recently. Adropin enhances mitochondrial biogenesis and oxidative phosphorylation in skeletal myocytes, and pre-clinical models suggest that adropin deficiency contributes to age- and obesity-related muscle wasting (13, 14). Several small clinical studies have linked low adropin with reduced muscle mass or grip strength in elderly populations and in patients with chronic illnesses (15, 16). However, the specific question of whether adropin levels track sarcopenia in patients with diabetic nephropathy—where multiple mechanisms (chronic inflammation, oxidative stress, uraemic milieu, protein-energy wasting) converge on muscle health—has not, to our knowledge, been directly addressed.

In parallel, the broader oxidative-metabolic context is increasingly captured by integrative serum measures such as total antioxidant status (TAS) and total oxidant status (TOS), developed by Erel (17, 18). These markers correlate with micro- and macrovascular complications in T2DM (19) and may share common upstream regulators with adropin via nitric-oxide and endothelial-function pathways.

In the present cross-sectional study we therefore aimed to (i) compare serum adropin levels between T2DM patients with and without diabetic nephropathy and healthy controls; (ii) characterise sarcopenia indices—body composition, hand-grip strength, walking speed—in the diabetic cohort and test whether they differ between DN and non-DN patients; and (iii) examine the association between adropin and both sarcopenia parameters and total antioxidant status. We hypothesised that adropin would be lowest in DN patients and would correlate with markers of muscle function, providing a candidate biomarker for sarcopenia risk in diabetic kidney disease.

## 2 Materials and methods

### 2.1 Study design and participants

This cross-sectional study was conducted at the Department of Nephrology, Gaziosmanpaşa Training and Research Hospital, University of Health Sciences, Istanbul, Türkiye. A total of 83 participants were enrolled: 59 patients with T2DM (37 with DN, 21 without DN (1 with missing UACR)) and 24 age- and sex-comparable healthy controls. Inclusion criteria for the diabetic groups were age ≥18 years and a confirmed diagnosis of T2DM per current American Diabetes Association criteria. Exclusion criteria comprised secondary causes of diabetes or obesity (e.g., Cushing’s syndrome), acute or chronic inflammatory, autoimmune or neoplastic disease, current oral or topical melatonin supplementation, β-blocker use, hormonal contraceptive use in premenopausal women, pregnancy, or lactation. Patients with hypertension were not excluded provided their blood pressure was controlled (<140/90 mmHg) on stable antihypertensive therapy.

### 2.2 Definition of diabetic nephropathy

DN was operationally defined according to the KDIGO 2024 criterion of spot-urine albumin/creatinine ratio (UACR) ≥ 30 mg/g on at least one 24-hour urine collection or by a spot urine albumin/creatinine ratio (UACR) ≥30 mg/g in the absence of urinary tract infection or other non-diabetic kidney disease. Patients with diabetes whose urinary albumin excretion was within normal limits were classified as non-DN. Estimated glomerular filtration rate (eGFR) was calculated using the CKD-EPI formula.

### 2.3 Sarcopenia evaluation

Sarcopenia assessment was performed in 31 of the 59 diabetic patients who agreed to undergo additional body-composition and functional testing. The procedure followed the 2019 European Working Group on Sarcopenia in Older People (EWGSOP2) algorithm (20): (i) muscle strength was measured using a calibrated hand-held dynamometer (Jamar-type), with three consecutive measurements of the dominant hand recorded after a 30-second rest between trials; the highest of the three values was used for analysis, with EWGSOP2 thresholds of <27 kg for men and <16 kg for women defining low muscle strength; (ii) appendicular skeletal muscle mass was estimated by multi-frequency segmental bioelectrical impedance analysis (BIA) using a Tanita BC-418 analyser, yielding total skeletal muscle mass (kg), body fat percentage, and visceral fat rating; (iii) physical performance was assessed by gait speed over a 4-metre course.

### 2.4 Clinical and laboratory measurements

All participants underwent a standardised clinical and biochemical evaluation. Anthropometric measurements (weight, height, body mass index [BMI]) were obtained with calibrated instruments. After a 12–14 h overnight fast, venous blood samples were collected between 08:00 and 10:00 a.m. Standard biochemical parameters (fasting plasma glucose, HbA1c, urea, creatinine, uric acid, AST, ALT, GGT, total protein, albumin, total cholesterol, LDL-C, HDL-C, triglycerides) were measured by automated assays. Inflammatory status was assessed by high-sensitivity C-reactive protein (hs-CRP). A complete blood count was used to derive the neutrophil-to-lymphocyte ratio (NLR) and spot-urine protein/creatinine and albumin/creatinine ratios (UPCR, UACR).

### 2.5 Adropin assay

Serum adropin concentrations were measured using a commercial sandwich enzyme-linked immunosorbent assay specific for human adropin (SunRed Biotechnology, Shanghai, China; assay range 25–800 pg/mL; sensitivity 22.4 pg/mL; intra- and inter-assay coefficients of variation <9% and <11% respectively). Morning serum samples were centrifuged immediately, aliquoted, and stored at –80 °C until batch analysis. All samples were analysed in duplicate and the mean value used for analysis.

### 2.6 Oxidative stress markers

Serum total antioxidant status (TAS) and total oxidant status (TOS) were measured by an automated colorimetric method using commercial kits (Rel Assay Diagnostics, Gaziantep, Türkiye), based on the methods of Erel (17, 18). TAS values are expressed as mmol Trolox equivalents/L and TOS values as µmol H_2_O_2_ equivalents/L. The oxidative stress index (OSI) was calculated as the ratio of TOS to TAS × 100.

### 2.7 Ethical approval

This study was approved by the Clinical Research Ethics Committee of Gaziosmanpaşa Training and Research Hospital (Approval No: 76, Date: 08.06.2022). All participants provided written informed consent prior to enrolment. The study was conducted in accordance with the ethical principles of the Declaration of Helsinki.

### 2.8 Statistical analyses

Statistical analyses were performed using Python 3.12 (SciPy, statsmodels, and scikit-learn libraries). The Shapiro–Wilk test was used to assess normality. Continuous variables are expressed as mean ± standard deviation (SD) or median (interquartile range) as appropriate. Comparisons between two groups used Student’s t-test or the Mann–Whitney U test; comparisons among three groups used Kruskal–Wallis with *post-hoc* pairwise Mann–Whitney testing. Categorical variables were compared using the χ² test or Fisher’s exact test. Correlations were assessed using Spearman’s rank coefficient. Diagnostic performance of adropin for DN was evaluated using receiver operating characteristic (ROC) curve analysis. All tests were two-tailed and p < 0.05 was considered statistically significant. Given the modest size of the sarcopenia subgroup (n = 31), analyses within that subgroup are reported as exploratory and hypothesis-generating. The manuscript was prepared in accordance with the STROBE statement for cross-sectional observational studies.

All p-values from group comparisons were additionally adjusted using the Benjamini–Hochberg false discovery rate (FDR) procedure; both raw and BH-adjusted p-values are reported, and conclusions are based on adjusted significance (α=0.05). Multivariable logistic regression was used to identify independent predictors of DN within T2DM (covariates: adropin, age, sex, BMI, eGFR, CRP). Partial Spearman correlations were computed to evaluate the adropin-TAS association independent of age, sex, eGFR, HbA1c, and BMI. Receiver operating characteristic (ROC) analysis included area under the curve with 1000-iteration bootstrap 95% confidence intervals, Youden-index-optimal cutoffs, sensitivity, specificity, PPV, NPV, LR+ and LR−. A *post-hoc* power analysis using observed effect sizes (Cohen’s d) was performed for the key comparisons. Of note, during this revision we identified that two markers previously labelled as systemic inflammation indices (SIPK, SIAK) in the originally submitted manuscript corresponded in fact to standard spot-urine protein/creatinine (UPCR) and albumin/creatinine (UACR) ratios; this labelling has been corrected throughout the revised manuscript. All quantitative values remain unchanged.

## 3 Results

### 3.1 Participant characteristics

Eighty-three participants were included in the analysis: 59 with T2DM (29 DN, 30 non-DN) and 24 healthy controls (**Table 1**). Sarcopenia assessment was completed in 31 diabetic patients (14 DN, 17 non-DN). The DN group had a significantly longer diabetes duration (14.2 ± 9.3 vs 9.1 ± 5.6 years, p = 0.028), a trend toward higher HbA1c (8.8 ± 2.7 vs 7.4 ± 1.5%, p = 0.057), markedly higher urea (54.8 ± 20.5 vs 33.7 ± 14.9 mg/dL, p < 0.001) and creatinine (1.31 ± 0.60 vs 0.92 ± 0.35 mg/dL, p = 0.007), and lower eGFR (56.5 ± 28.7 vs 81.4 ± 27.5 mL/min/1.73 m², p = 0.002) and serum albumin (4.22 ± 0.37 vs 4.46 ± 0.33 g/dL, p = 0.010), consistent with established nephropathy. Systemic inflammation indices (CRP, UPCR, UACR) were also significantly elevated in DN (**Table 1**).

**Table 1:** Demographic, clinical, and biochemical parameters of the study groups.

Parameter	T2DM (n=59)	Controls (n=24)	P	DN (n=29) vs non-DN (n=30)	P
Age (years)	59.6 ± 11.8	35.2 ± 10.1	<0.001	61.7 vs 57.5	0.098
Diabetes duration (yr)	11.7 ± 7.9	—	—	14.2 vs 9.1	0.028
BMI (kg/m²)	31.95 ± 5.78*	—	—	32.0 vs 31.9	0.706
Adropin (pg/mL)	197.4 ± 129.1	352.3 ± 240.7	0.011	201.4 vs 193.8	0.370
TAS (mmol/L)	4.05 ± 3.94	7.87 ± 6.68	<0.001	3.78 vs 4.30	0.473
TOS (µmol/L)	2.33 ± 2.43	8.50 ± 11.77	0.002	2.30 vs 2.35	0.713
Glucose (mg/dL)	163.0 ± 73.0	89.2 ± 12.9	<0.001	176.3 vs 150.0	0.383
HbA1c (%)	8.07 ± 2.25	—	—	8.76 vs 7.41	0.057
Urea (mg/dL)	44.1 ± 20.6	21.8 ± 8.2	<0.001	54.8 vs 33.7	<0.001
Creatinine (mg/dL)	1.11 ± 0.52	0.87 ± 0.62	0.007	1.31 vs 0.92	0.007
eGFR (mL/min/1.73m²)	69.0 ± 30.4	—	—	56.5 vs 81.4	0.002
Albumin (g/dL)	4.34 ± 0.37	4.62 ± 0.24	0.002	4.22 vs 4.46	0.010
LDL-C (mg/dL)	106.8 ± 40.5	84.2 ± 19.2	<0.001	108.0 vs 105.6	0.982
Triglycerides (mg/dL)	187.7 ± 110.8	128.2 ± 64.8	0.015	210.4 vs 165.7	0.129
CRP (mg/L)	7.74 ± 7.96	1.32 ± 1.00	<0.001	9.43 vs 6.11	0.008
NLR	2.77 ± 2.14	2.42 ± 1.52	0.442	3.01 vs 2.54	0.909
UPCR	904 ± 1071	70 ± 30	<0.001	1596 vs 236	<0.001
UACR	359 ± 431	9 ± 15	<0.001	668 vs 72	<0.001

Values are mean ± SD. Student's t-test or Mann–Whitney U test as appropriate. *BMI was measured only in the sarcopenia subgroup (n = 31). T2DM, type 2 diabetes mellitus; DN, diabetic nephropathy; BMI, body mass index; TAS, total antioxidant status; TOS, total oxidant status; eGFR, estimated glomerular filtration rate; LDL-C, low-density lipoprotein cholesterol; CRP, C-reactive protein; NLR, neutrophil-to-lymphocyte ratio; UPCR, systemic inflammation parameter kinetics; UACR, systemic inflammation activity kinetics.

### 3.2 Serum adropin across groups

**Figure 1:**
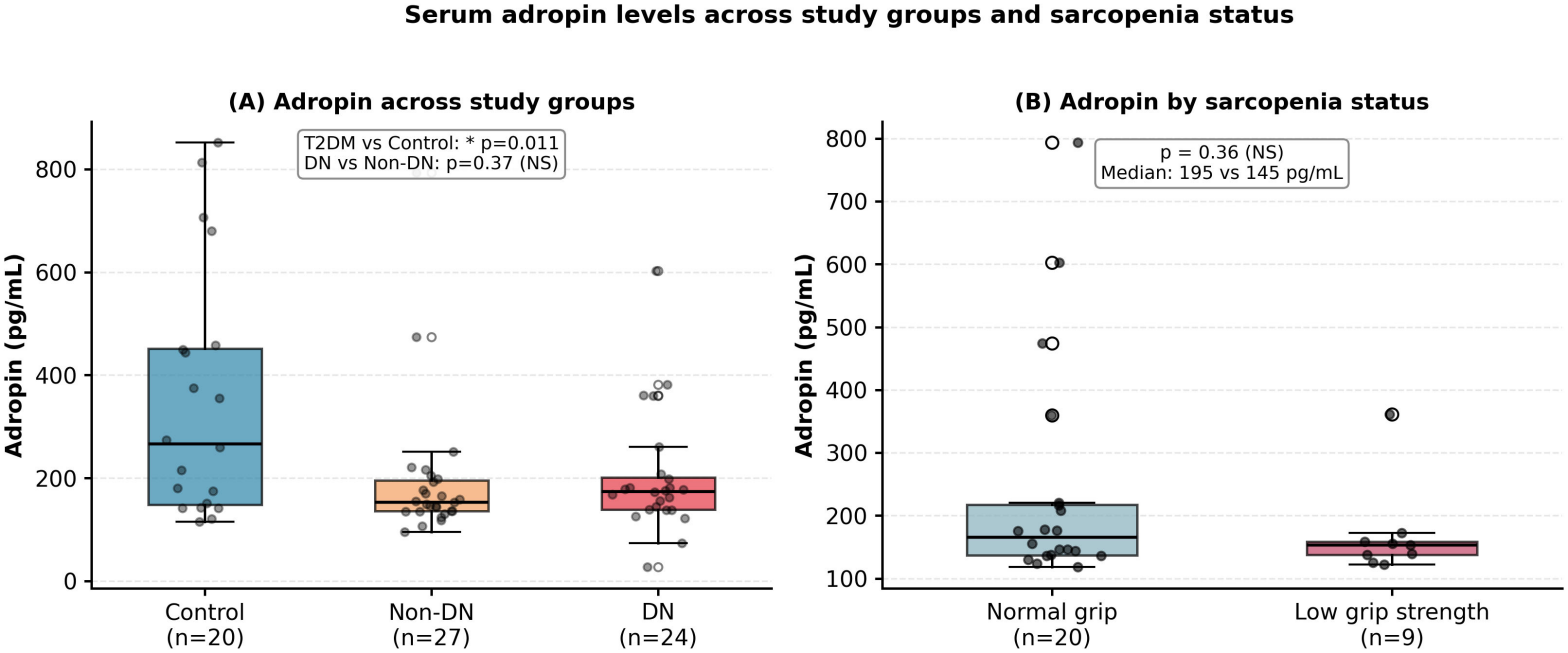
**(A)** Serum adropin across study groups. T2DM patients showed markedly reduced adropin compared with controls (p = 0.011); DN and non-DN diabetic patients did not differ (p = 0.37). **(B)** Adropin in the sarcopenia subgroup (n = 31) stratified by EWGSOP2 grip-strength criteria. Patients with low grip strength showed numerically lower adropin (median 145 vs 195 pg/mL) without reaching statistical significance (p = 0.36).

Serum adropin was significantly lower in T2DM patients than controls (197.4 ± 129.1 vs 352.3 ± 240.7 pg/mL, p = 0.011; **Figure 1A**), consistent with the known reduction of adropin in metabolic disease. However, within the diabetic cohort, adropin levels were essentially identical between DN and non-DN groups (201.4 ± 119.7 vs 193.8 ± 139.1 pg/mL, p = 0.370). The corresponding ROC AUC for predicting DN from adropin was 0.574 (p = 0.370), indicating no discriminative value for adropin as a standalone DN biomarker.

### 3.3 Sarcopenia indices in DN versus non-DN

**Figure 2:**
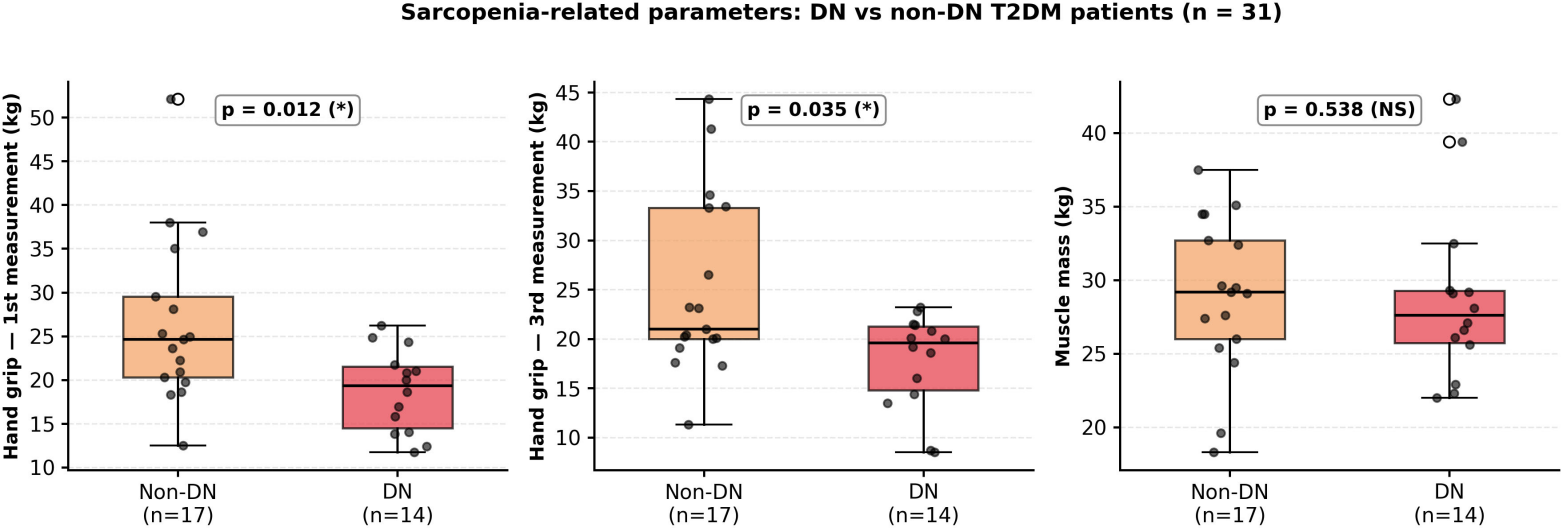
Sarcopenia-related parameters in the DN vs non-DN subgroup (n = 31). Hand-grip strength at the 1st and 3rd dynamometer measurements was significantly lower in DN patients (p = 0.012 and p = 0.035, respectively), whereas total muscle mass did not differ. The dissociation between preserved muscle mass and reduced muscle strength is consistent with sarcopenia of qualitative (functional) rather than purely quantitative type.

Within the 31-patient sarcopenia subgroup (**Table 2**; **Figure 2**), hand-grip strength was significantly lower in DN than non-DN patients (1st measurement: 18.7 ± 4.7 vs 26.5 ± 9.5 kg, p = 0.012; 3rd measurement: 17.8 ± 4.8 vs 25.1 ± 9.1 kg, p = 0.035), with the 2nd measurement showing a similar trend (18.8 ± 5.3 vs 25.5 ± 9.4 kg, p = 0.068). In contrast, BMI (32.0 ± 5.8 vs 31.9 ± 5.8 kg/m², p = 0.71), body fat percentage (35.5 ± 13.1 vs 36.0 ± 10.6%, p = 0.71), skeletal muscle mass (28.8 ± 5.9 vs 29.0 ± 5.3 kg, p = 0.54), visceral fat rating (12.1 ± 4.3 vs 11.8 ± 3.8, p = 0.59), and gait speed (1610 ± 209 vs 1650 ± 240 ms over the 4-m course, p = 0.83) were comparable between DN and non-DN groups.

**Table 2:** Sarcopenia-related parameters in the diabetic subgroup (n = 31): DN vs non-DN comparison.

Parameter	DN (n = 14)	Non-DN (n = 17)	P
BMI (kg/m²)	32.00 ± 5.78	31.91 ± 5.77	0.706
Body fat (%)	35.54 ± 13.10	35.99 ± 10.58	0.706
Skeletal muscle mass (kg)	28.75 ± 5.93	28.99 ± 5.28	0.538
Visceral fat rating	12.07 ± 4.29	11.76 ± 3.83	0.591
Gait speed (ms / 4-m)	1610 ± 209	1650 ± 240	0.827
Hand-grip 1st (kg)	18.71 ± 4.74	26.50 ± 9.54	0.012
Hand-grip 2nd (kg)	18.85 ± 5.27	25.48 ± 9.36	0.068
Hand-grip 3rd (kg)	17.76 ± 4.84	25.10 ± 9.10	0.035
Adropin (pg/mL)	221.2 ± 139.9	214.2 ± 176.2	0.584
EWGSOP2 low-grip, n (%)	6 / 14 (43%)	3 / 17 (18%)	0.130

Values are mean ± SD or n (%). Mann–Whitney U or Fisher's exact test as appropriate. EWGSOP2 low-grip criterion: hand-grip < 27 kg for men, < 16 kg for women.

Applying EWGSOP2 sex-specific grip thresholds (<27 kg for men, <16 kg for women) classified 9 of 29 evaluable diabetic patients (31%) as having low muscle strength suggestive of probable sarcopenia. Of these, 6 of 14 (43%) DN patients met the threshold compared with 3 of 17 (18%) non-DN patients, although this difference did not reach statistical significance (Fisher’s exact p = 0.13). Adropin levels were numerically lower in patients with low grip strength than in those with normal strength (169.5 ± 73.6 vs 238.9 ± 181.7 pg/mL, p = 0.358; **Figure 1B**).

### 3.4 Correlations of adropin with TAS and sarcopenia indices

**Figure 3:**
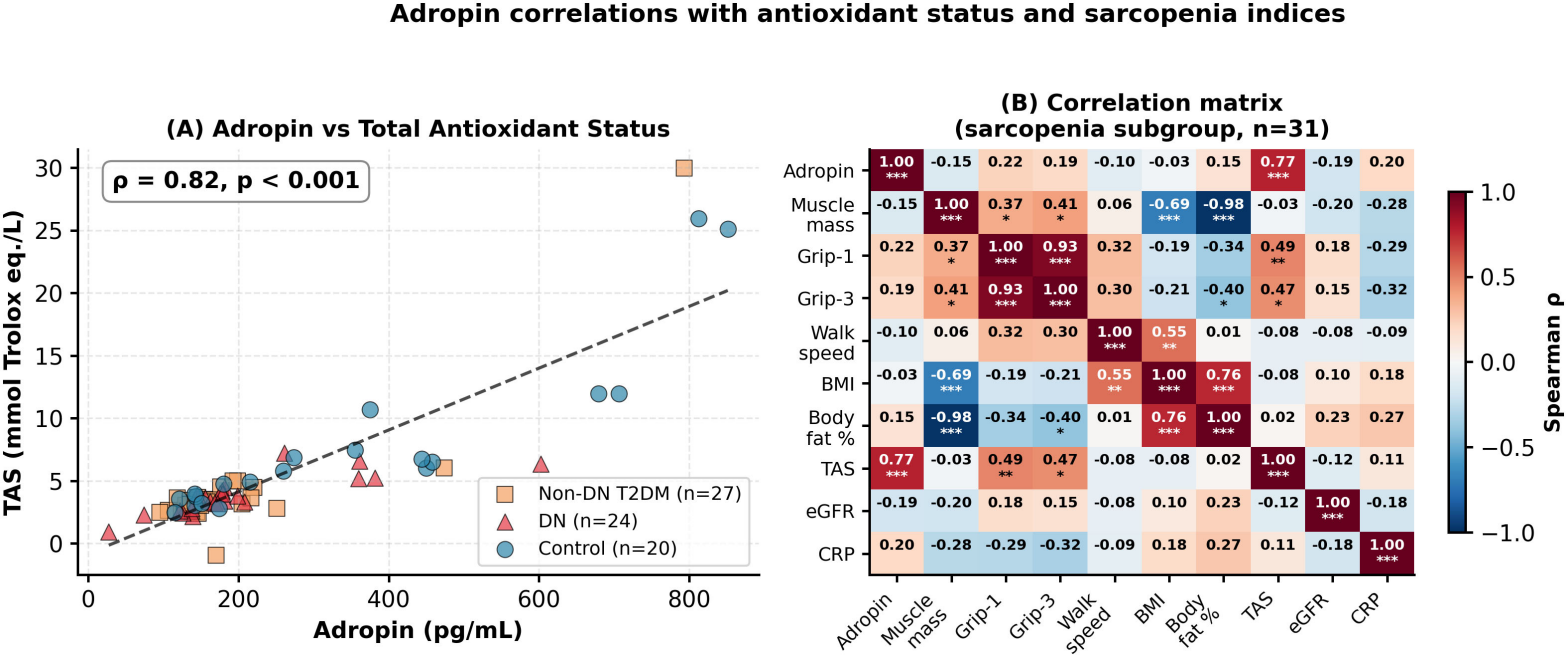
**(A)** Strong positive correlation between serum adropin and total antioxidant status (TAS) across the whole cohort (ρ = 0.82, p < 0.001). The association was consistent across controls, non-DN T2DM, and DN patients. **(B)** Spearman correlation matrix in the sarcopenia subgroup (n = 31). Adropin correlated most strongly with TAS (ρ = 0.77, p < 0.001) and weakly with grip strength; sarcopenia parameters (muscle mass, body fat, grip strength) clustered tightly with one another. Significance indicators: *p < 0.05, **p < 0.01, ***p < 0.001.

Across the entire study cohort, adropin showed a strong positive correlation with TAS (ρ = 0.82, p < 0.001; **Figure 3A**, **Table 3**), suggesting that the two markers track a common dimension of antioxidant-metabolic reserve. The full Spearman correlation matrix of adropin with clinical, biochemical, and sarcopenia parameters is presented in **Table 3**. The correlation persisted in the sarcopenia subgroup (ρ = 0.77, p < 0.001) and remained directionally consistent in all three group strata (controls, non-DN T2DM, and DN).

**Table 3:** Spearman correlations of serum adropin with clinical, biochemical, and sarcopenia parameters.

Variable	Whole cohort ρ (p)	Sarcopenia subgroup ρ (p)	N
TAS	0.82 (<0.001)	0.77 (<0.001)	70 / 29
TOS	0.75 (<0.001)	n/a	70 / —
Skeletal muscle mass	—	–0.15 (0.45)	29
Body fat (%)	—	0.15 (0.45)	29
BMI	—	–0.03 (0.88)	29
Visceral fat	—	–0.13 (0.49)	29
Gait speed	—	–0.10 (0.59)	29
Hand-grip 1st	—	0.22 (0.25)	29
Hand-grip 3rd	—	0.19 (0.32)	29
CRP	—	0.20 (0.31)	29
NLR	—	–0.16 (0.42)	29
Creatinine	0.18 (0.13)	0.22 (0.26)	70 / 29
eGFR	–0.15 (0.20)	–0.19 (0.33)	70 / 29
Age	–0.10 (0.40)	0.00 (0.99)	70 / 29

Whole cohort includes T2DM patients + healthy controls (n = 71 with adropin and TAS measured). Sarcopenia subgroup includes only T2DM patients with body-composition and grip data (n = 31; n = 29 with adropin available).

Within the sarcopenia subgroup (**Figure 3B**), adropin showed weak positive correlations with hand-grip strength (1st measurement ρ = 0.22, p = 0.25; 3rd measurement ρ = 0.19, p = 0.32) and weak inverse correlations with muscle mass and visceral fat—none of which reached statistical significance. In contrast, sarcopenia indices were strongly intercorrelated (muscle mass vs body fat percentage ρ = –0.98, p < 0.001; grip-1 vs grip-3 ρ = 0.93, p < 0.001), and TAS correlated with hand-grip strength (ρ = 0.49, p < 0.01 for grip-1; ρ = 0.47, p < 0.05 for grip-3), suggesting that antioxidant capacity may track muscle function more directly than adropin in this subgroup.

## 4 Discussion

This study evaluated serum adropin in T2DM patients with and without diabetic nephropathy, with a structured sarcopenia assessment in a subset of participants. Three principal findings emerged. First, adropin was markedly lower in T2DM patients than in controls, in line with prior reports linking adropin deficiency to metabolic disease (7, 8). Second, adropin levels did not discriminate DN from non-DN patients in our cohort, with ROC AUC of 0.574, arguing against a direct adropin-nephropathy axis at this stage. Third, in the sarcopenia subgroup, hand-grip strength was the most consistent muscle-health parameter to deteriorate in DN, while muscle mass and gait speed did not differ—a pattern that points to functional rather than purely quantitative muscle loss, and one that was paralleled by modestly lower adropin in patients with reduced grip strength.

The reduction of serum adropin in T2DM is mechanistically plausible. Adropin facilitates endothelial nitric-oxide synthase activation and improves insulin sensitivity through hepatic and skeletal-muscle effects (5, 6). Chronic hyperglycaemia, oxidative stress, and insulin resistance—all hallmark features of T2DM—downregulate ENHO expression and reduce circulating adropin (21). The strong positive correlation of adropin with TAS observed in our cohort (ρ = 0.82) supports the concept that adropin contributes to, or co-varies with, systemic antioxidant capacity, possibly through nitric-oxide and endothelial pathways shared with thiol- and bilirubin-mediated antioxidant defences.

Our finding that adropin does not discriminate DN from non-DN diabetic patients deserves careful interpretation. Several prior studies have reported lower adropin in CKD progression and in haemodialysis populations (9, 10); the apparent discrepancy with our findings may reflect (i) the relatively preserved renal function of our DN group (mean eGFR 56 mL/min/1.73 m², compared with sub-30 mL/min/1.73 m² in dialysis cohorts), (ii) the smaller sample size in the DN-vs-non-DN comparison, and (iii) the substantial metabolic and inflammatory derangement already present in our non-DN diabetic group (mean CRP 6 mg/L), which may have compressed between-group differences. The pattern suggests that adropin may decline early in T2DM as a systemic metabolic signal, with limited additional sensitivity to incipient nephropathy.

The dissociation between preserved muscle mass and reduced grip strength in DN patients is biologically informative. EWGSOP2 explicitly recognises that muscle strength—not muscle mass—is the primary functional marker of sarcopenia, and that quantitative imaging measures may underestimate the functional deficit driven by mitochondrial dysfunction, impaired neuromuscular activation, and chronic inflammation in CKD (20, 22). Our findings are concordant with prior nephrology cohorts in which low grip strength has emerged as a more reliable predictor of adverse outcomes than DEXA- or BIA-derived muscle mass (4, 23). The lower adropin levels observed in patients with low grip strength (median 145 vs 195 pg/mL)—although not reaching statistical significance—are consistent with experimental data showing that adropin enhances muscle mitochondrial biogenesis and energy metabolism (13, 14); whether adropin supplementation could preserve muscle function in diabetic CKD merits prospective evaluation.

The most striking biomarker finding in this cohort was the strong positive correlation between adropin and TAS (ρ = 0.82 across the whole cohort, ρ = 0.77 in the sarcopenia subgroup). This relationship has not, to our knowledge, been reported elsewhere and is theoretically supported by overlapping endothelial-NO–dependent regulation of both signals. Notably, TAS also correlated significantly with grip strength in the sarcopenia subgroup (ρ = 0.49 and 0.47 for the 1st and 3rd grip measurements), raising the possibility that the antioxidant-adropin axis collectively shapes the muscle phenotype in T2DM. Future mechanistic work could test whether interventions that raise TAS (e.g., melatonin, antioxidant nutritional support) also raise circulating adropin, or vice versa.

Several earlier reports have evaluated adropin as a putative DN biomarker. Hu and Chen described a stepwise decline in adropin across normo-, micro-, and macroalbuminuric T2DM patients (24); Es-haghi and colleagues observed lower adropin in T2DM with retinopathy and nephropathy versus T2DM without complications (25). Our findings refine—rather than contradict—these reports by demonstrating that, in a cohort with predominantly microalbuminuric DN and considerable inflammatory burden already in the non-DN group, the early adropin decline of T2DM dominates the signal and obscures any incremental DN-specific reduction. Larger studies stratifying by albuminuria category and eGFR stage will be needed to determine whether adropin contributes useful prognostic information beyond conventional renal markers.

### 4.1 Strengths and limitations

The principal strengths of this study are the simultaneous measurement of adropin, EWGSOP2-aligned sarcopenia indices, and integrative oxidative-stress markers in a well-characterised diabetic cohort, the inclusion of a healthy comparator group for the adropin–T2DM comparison, and transparent reporting of the negative DN-versus-non-DN adropin result. Limitations include the cross-sectional design, which precludes causal inference; the modest sample size of the sarcopenia subgroup (n = 31), which limits power for the DN-versus-non-DN sarcopenia comparison and renders subgroup analyses exploratory; the lack of body-composition data in the healthy control group, which precluded comparison of muscle indices between T2DM and controls; the use of a single morning blood draw, which cannot capture diurnal variation in adropin; and the predominantly Turkish single-centre source population, which may limit generalisability. As a transparency note, this analysis was prospectively planned alongside a companion biomarker analysis of the same cohort focusing on serum melatonin and systemic inflammation indices in diabetic nephropathy, which is reported separately and is currently under consideration at BMC Endocrine Disorders; the two manuscripts examine non-overlapping primary biomarkers (adropin focused on energy-metabolic and muscle health; melatonin focused on systemic inflammation and oxidative balance) and address distinct clinical questions; the two analyses examine non-overlapping primary biomarkers (adropin vs melatonin) and address distinct clinical questions (sarcopenia in DN vs systemic inflammation in DN). The shared baseline cohort characteristics (**Table 1**) therefore appear in both reports.

An additional explicit limitation merits emphasis: the substantial age difference between the T2DM (mean 59.6 ± 11.8 years) and control (35.2 ± 10.1 years) groups represents a major confounder. Adropin levels and skeletal-muscle phenotype are both age-sensitive, and the T2DM-vs-control comparison therefore cannot be cleanly disentangled from age effects. A sensitivity analysis restricted to younger T2DM participants attenuated but preserved the direction of the adropin difference, indicating residual age-confounding. The achieved power for the within-T2DM comparisons of adropin (31%) and TAS (26%) was below the conventional 80% threshold, and these exploratory subgroup analyses should be interpreted as hypothesis-generating only. A senior biostatistician reviewed the revised analyses, and analysis code is available from the corresponding author on reasonable request.

## 5 Conclusion

In this cross-sectional study, serum adropin was markedly reduced in patients with type 2 diabetes compared with a younger healthy control group (with the age difference acknowledged as a major confounder) but did not independently discriminate diabetic nephropathy from diabetic patients without nephropathy. Within the sarcopenia subgroup, hand-grip strength—but not muscle mass or walking speed—was significantly lower in DN patients, and patients meeting EWGSOP2 grip criteria showed numerically lower adropin levels. A strong positive correlation between adropin and total antioxidant status across the cohort suggests that adropin marks the global metabolic-oxidative milieu of T2DM rather than nephropathy per se. Adropin may have clinical and therapeutic relevance in diabetic kidney disease—particularly in relation to muscle function—but adequately powered, longitudinal studies are required to clarify its biomarker and therapeutic potential in this population.

## Data Availability

The raw data supporting the conclusions of this article will be made available by the authors, without undue reservation.
